# Proteomic profiling of eIF3a conditional knockout mice

**DOI:** 10.3389/fmolb.2023.1160063

**Published:** 2023-04-19

**Authors:** Wei Zhuo, Juan Chen, Shilong Jiang, Juyan Zheng, Hanxue Huang, Pan Xie, Wei Li, Mengrong Lei, Jiye Yin, Ying Gao, Zhaoqian Liu

**Affiliations:** ^1^ Department of Clinical Pharmacology, Hunan Key Laboratory of Pharmacogenetics, National Clinical Research Center for Geriatric Disorders, Xiangya Hospital, Central South University, Changsha, China; ^2^ Engineering Research Center for Applied Technology of Pharmacogenomics of Ministry of Education, Institute of Clinical Pharmacology, Central South University, Changsha, China; ^3^ Departments of Pharmacy, Xiangya Hospital, Central South University, Changsha, China; ^4^ Departments of Gerontology, Xiangya Hospital, Central South University, Changsha, China

**Keywords:** eIF3a, knockout mice, oxidative stress, proteomics, protein landscape

## Abstract

Eukaryotic translation initiation factor 3 subunit A (eIF3a) is the largest subunit of the eukaryotic translation initiation factor 3 (eIF3). eIF3a plays an integral role in protein biosynthesis, hence impacting the onset, development, and treatment of tumors. The proteins regulated by eIF3a are still being explored *in vivo*. In this study, a Cre-loxP system was used to generate eIF3a conditional knockout mice. Tandem mass tag (TMT) labeling with LC-MS/MS analysis was used to identify differentially expressed proteins (DEPs) in fat, lungs, skin, and spleen tissue of the eIF3a knockout mice and controls. Bioinformatics analysis was then used to explore the functions and molecular signaling pathways of these protein landscapes. It was observed that eIF3a is essential for life sustenance. Abnormal tissue pathology was found in the lungs, fat, skin, spleen, and thymus. In total, 588, 210, 324, and 944 DEPs were quantified in the lungs, fat, skin, and spleen, respectively, of the eIF3a knockout mice as compared to the control. The quantified differentially expressed proteins were tissue-specific, except for eight proteins shared by the four tissues. A broad range of functions for eIF3a, including cellular signaling pathway, immune response, metabolism, defense response, phagocytes, and DNA replication, has been revealed using bioinformatics analysis. Herein, several pathways related to oxidative stress in the Kyoto Encyclopedia of Genes and Genomes (KEGG) database, including nitrogen metabolism, peroxisome, cytochrome P450 drug metabolism, pyruvate metabolism, PPAR signaling pathway, phospholipase D signaling pathway, B-cell receptor signaling pathway, ferroptosis, and focal adhesion, have been identified. Collectively, this study shows that *eIF3a* is an essential gene for sustaining life, and its downstream proteins are involved in diverse novel functions beyond mRNA translational regulation.

## 1 Introduction

Transcription and translation are two crucial stages of gene expression and regulation. Transcription is the process of transforming genetic information from DNA to RNA, whereas translation is the process of converting mRNA to amino acids. Translation is the most critical process in gene regulation (Schwanhausser et al., 2011); it includes initiation, extension, termination, and ribosome recycling steps. The initiation step of mRNA translation is rate-limiting during protein synthesis (Sonenberg and Hinnebusch, 2009). In eukaryotes, 12 factors are identified during translation, and eukaryotic translation initiation factor 3 (eIF3) is the largest and most complex factor comprising 13 subunits (eIF3a–eIF3m). Eukaryotic translation initiation factor 3 subunit A (eIF3a) is the largest subunit (Yin et al., 2018), and it plays a crucial role in both cap-dependent and cap-independent translations (Saletta et al., 2010; Yin et al., 2013; Meyer et al., 2015). A structure study (Wagner et al., 2016) revealed that eIF3a, one of the core elements in eIF3, with other subunits of eIFs, plays a vital role in the regulation of global protein translation (Methot et al., 1997; Dong et al., 2013; Aylett et al., 2015). In addition, eIF3a was involved in cell cycle arrest, cell apoptosis, cell proliferation (Yin et al., 2018), embryo development (Liu et al., 2007), virus infection (Buratti et al., 1998; Rodriguez Pulido et al., 2007; Wang et al., 2012; Subramani et al., 2018), promotion of tumor initiation and development, and chemotherapy and radiation therapy of cancers (Yin et al., 2011a; Yin et al., 2018). High-throughput sequencing revealed that eIF3a is associated with estrogen receptor response (Yamaga et al., 2013), diabetes (Carty et al., 2014; Jin et al., 2014), and immune response (Mao et al., 1992; Kroczynska et al., 2009; Cheng et al., 2016). Furthermore, eIF3a expression is a response to stress (Dong et al., 2009; Lane et al., 2013; Malcova et al., 2021; Shu et al., 2022). However, whether *eIF3a* was an oxidative stress-related gene remains uncertain.

Unveiling the downstream proteins of eIF3a, a critical translational initiation factor in protein translation, is the core activity to elucidate the molecular mechanism and explore new functions. It was speculated that eIF3a regulates 15%–20% of proteins *in vitro* (Dong and Zhang, 2003). In contrast, eIF3a does not significantly affect translation initiation activity in reticulocyte (Chaudhuri et al., 1997). Currently, there are neither animal models specifically designed to investigate *eIF3a* gene nor studies aimed at exploring the downstream proteins regulated by this gene *in vivo*. In the last few decades, studies on eIF3a in relation to cancer have revealed that eIF3a is an oncogene in different cancers, which correlates with the prognosis and affects cisplatin, anthracycline, vemurafenib, irinotecan, and radiotherapy treatment (Yin et al., 2011b; Xu et al., 2013; Zhang et al., 2015; Fang et al., 2017; Tumia et al., 2020; Chen J et al., 2021; Chen Y X et al., 2021; Jiang et al., 2021; Mei et al., 2022a; Mei et al., 2022b), especially cisplatin and radiation, two indispensable means of cancer treatment, which have been widely proven to promote oxidative stress (Cepeda et al., 2007; Sisakht et al., 2020). It is suggested that eIF3a may be involved in a new field-oxidative stress. As a crucial translation initiation factor, what has already been discovered is only the tip of the iceberg. Numerous unexplored functions await to be unveiled.

Previously, we found that the expression of eIF3a is time-dependent, which is high in the embryonic period and decreases in the post-birth period (Liu et al., 2007), suggesting that eIF3a plays an essential role in embryonic development. Herein, the Cre-LoxP system, a powerful tool for the spatiotemporal control of gene expression, was used to generate an eIF3a conditional knockout animal model for addressing the function of eIF3a *in vivo*. Tandem mass tag (TMT) systems and bioinformatics were used to unveil the protein expression profile in eIF3a-deficient mice, characterize the differentially expressed proteins (DEPs), and explore new functions and mechanisms of eIF3a.

## 2 Materials and methods

### 2.1 Chemicals and reagents

Chemicals and reagents used in this study were purchased from the following sources: corn oil and tamoxifen from Aladdin (Shanghai, China); Taq Plus Master Mix Ⅱ, GelRed Nucleic Acid Stain, agarose gel, DNA markers, and DNA Isolation Kit from Vazyme (Nanjing, China); total RNA extraction reagent, RT-PCR kit, and SYBR Premix Ex Taq™ kit from Takara (Dalian, China); eIF3a and GAPDH antibody from Abcam (Cambridge, UK); methanol, ethanol, acetone, and acetonitrile from Sinopharm (Shanghai, China); and BCA Protein Assay Kit from Invitrogen (Grand Island, United States).

### 2.2 Establishment of eIF3a conditional knockout mice

**FIGURE 1:**
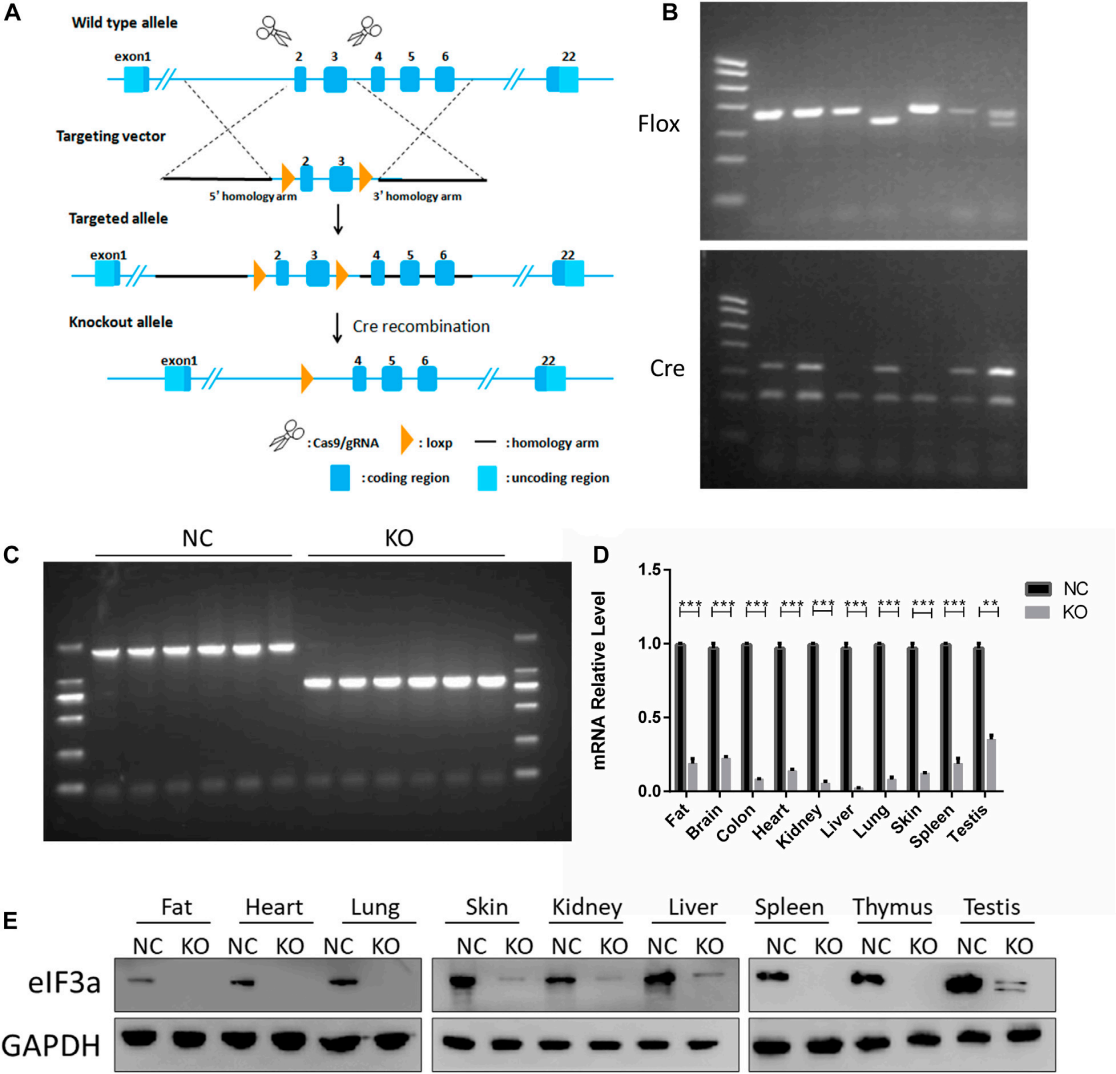
Generation and validation of eIF3a conditional knockout mouse. **(A)** Strategy for eIF3a knockout mice. Exons 2–3 of eIF3a were flanked with two floxP sites (triangles). Exons 2–3 were deleted by Cre/floxP recombination, producing the eIF3a knockout allele. **(B)** Representative genotyping results of flox and Cre obtained by PCR using tail DNA. **(C)** Representative genotyping results obtained by PCR using tail DNA from the control (1914bp) and eIF3a knockout mice (835 bp), following tamoxifen administration. **(D)** mRNA of eIF3a in different tissues of control and eIF3a knockout mice. **p < 0.01, ***p < 0.001; N = 6. **(E)** Immunoblot analysis of eIF3a levels in different organs of control and eIF3a knockout mice.

Mice were housed in high-efficiency particulate air (HEPA)-filtered cages in a specified pathogen-free environment on a 12-h/12-h light/dark cycle. Food and water were made available *ad libitum*. All experimental procedures were audited and approved by the Animal Ethics Committee of Xiangya Hospital. The Cre-LoxP system was used in C57BL/6 mice to generate eIF3a conditional knockout mice. The eIF3a-floxed mice were developed by inserting floxP sites between exons 2 and 3 (Figure 1A) at the Shanghai Model Organism Center. Briefly, Cas9 mRNAs, gRNAs (gRNA1 TGT​GTT​TAT​GTA​GAG​GGA​CTC​GG, gRNA2 CCA​TCC​ATG​ATT​TCA​GTT​TCT​GG), and homologous recombination vectors (Supplementary Figure S1) were microinjected into the fertilized eggs of C57BL/6J mice to obtain F0-generation mice. Chimeric mice were mated with wild-type mice to obtain eIF3a^flox/+^ mice, and then, eIF3a^flox/+^ and eIF3a^flox/+^ mice were bred to obtain eIF3a^flox/flox^ mice. UBC-Cre-ERT2 transgenic mice (from the Jackson Laboratory) were bred with eIF3a^flox/flox^ mice to remove the floxP-flanked exons in all tissues following the induction of tamoxifen. Finally, the mice model carrying two floxed eIF3a alleles and those are Cre-positive (eIF3a^flox/flox^; Cre), were selected, whereas those carrying two floxed eIF3a alleles but lacking Cre (eIF3a^flox/flox^) were used as a control. At the 8th week, all mice were administered with tamoxifen (75 mg/kg) *via* intraperitoneal injections once daily for five days. The mice were euthanized, and then, blood and tissues were collected 24 h after the last dose. Immediately, one portion of the tissues was fixed in 4% paraformaldehyde; the others were frozen in liquid nitrogen.

### 2.3 DNA extraction and PCR gel electrophoresis

DNA was extracted according to the manufacturer’s protocols. The polymerase chain reactions (PCRs) were performed using MJ Research Thermal Cyclers. PCR products were separated by electrophoresis on 2% agarose gel and imaged using the ChemiDoc Imaging System (Bio-Rad, United States). Primers are listed in Supplementary Table S1.

### 2.4 Serum biochemistry detection

Serum biochemical indices were detected using an automated biochemical analyzer.

### 2.5 RNA extraction and real-time qPCR

Total RNA from tissues was extracted using TRIzol reagent according to the manufacturer’s instructions. A volume of 1 μg total RNA was used to synthesize cDNA using the reverse transcriptase reagent kit as per the manufacturer’s instruction. Real-time qPCR reactions were carried out using SYBR Premix Ex Taq™ in the LightCycler 480 System (Roche, Swiss). The relative expression of the eIF3a was calculated using the 2^−ΔΔCT^ method. The primer list is provided in Supplementary Table S1.

### 2.6 Histological staining and immunofluorescence

The organs fixed in 4% paraformaldehyde were embedded in paraffin. Briefly, the paraffin-embedded tissues (serial 5-μm sections) were stained with hematoxylin and eosin (H&E) for morphological analysis using a microscope. After antigen retrieval, the sections were incubated with primary antibodies at 4°C overnight, and then, they were incubated with secondary antibodies for 2 h at room temperature. Slides were prepared using DAPI and observed using a fluorescence microscope.

### 2.7 Western blotting analysis

The tissues were subjected to lysis on ice for 30 min using RIPA buffer. The lysis buffer was supplemented with a protease inhibitor. The lysed mixture was centrifuged at ×12,000 g for 15 min at 4°C. The protein concentration of the supernatant was determined by BCA assay. The proteins were resolved on SDS-PAGE gels and later transferred to PVDF membranes (0.45 µm). Post transfer, the PVDF membranes were blocked with skim milk and incubated overnight at 4°C with respective antibodies in 5% BSA. This was followed by incubation with a secondary antibody at room temperature for 1 h. Finally, the protein signals were detected using an enhanced chemiluminescence kit.

### 2.8 TMT labeling and HPLC fraction

Proteins were extracted from fat, lungs, skin, and spleen of eIF3a knockout and control mice. Briefly, proteins were treated in 5 mM dithiothreitol and alkylated in 11 mM iodoacetamide; lastly, the protein solution was digested using trypsin. Afterward, peptides were desalted using the Strata X C18 SPE column and vacuum-dried. The peptides were labeled with TMT according to the manufacturer’s protocol. Briefly, one unit of the TMT reagent was mixed with peptides; then, the mixture was incubated for 2 h at room temperature, and lastly, it was desalted and dried. The peptides were fractionated by high-performance liquid chromatography (HPLC) using the Thermo BetaSil C18 column (5 μm particles, 10 mm ID, 250 mm length). Briefly, the peptides were separated with gradient acetonitrile (8%–32%) into 60 fractions; then, they were combined and dried by vacuum centrifugation.

### 2.9 LC-MS/MS analysis and data search

The peptides were separated on an EASY-nLC 1000 ultra-high performance liquid phase system (UHPLC). Subsequently, they were ionized in an NSI ion source and analyzed by HF-X mass spectrometry. The peptide precursor ions and their secondary fragments were detected and analyzed using a high-resolution Orbitrap. The m/z scan ranged from 350 to 1800 for full scan, and intact peptides were detected in the Orbitrap at a resolution of 70,000. The peptides were then selected for MS/MS using the NCE setting as 28, and the fragments were detected in the Orbitrap at a resolution of 17,500. A data-dependent procedure was employed, which alternated between one MS scan followed by 20 MS/MS scans with 15.0 s dynamic exclusion. Automatic gain control (AGC) was set at 5E4, and the fixed first mass was set at 100 m/z.

### 2.10 Database search

The MS/MS data were processed using the MaxQuant search engine (v.1.5.2.8). Tandem mass spectra were searched in the UniProt database. Trypsin/P was specified as a cleavage enzyme allowing up to two missing cleavages. Mass tolerance for precursor ions was set at 20 ppm in the first search and 5 ppm in the main search, whereas mass tolerance for fragment ions was set at 0.02 Da. FDR was adjusted to <1%, and a minimum score for the peptides was set at >40. Screening of differentially expressed proteins (N = 3; *p* < 0.05; |FC| > 1.3 or <1/1.3) was performed prior to bioinformatics analysis.

### 2.11 Bioinformatics analysis

DEPs were subjected to multiple bioinformatics analysis. Gene Ontology (GO), annotation, and Kyoto Encyclopedia of Genes and Genomes (KEGG) pathways and enrichment analysis were conducted using the Database for Annotation, Visualization, and Integrated Discovery (DAVID) bioinformatics resources (https://david.ncifcrf.gov). *p* < 0.05 was considered significant.

### 2.12 Statistics

The Kaplan–Meier survival curve was generated using a log-rank test. Statistical analyses were performed by Student’s two-tailed *t*-test. *p*-values less than 0.05 were considered significant and labeled with * (*p < 0.05, **p < 0.01, and ***p < 0.001).

## 3 Results

### 3.1 Generation and identification of eIF3a knockout mice

The design of eIF3a knockout mice using the Cre-loxP system is presented in Figure 1A. Homozygous flox and Cre-positive mice were observed by DNA PCR gel electrophoresis (Figure 1B). A recombinant DNA band (835 bp) was observed in the eIF3a^flox/flox^, UBC-Cre-ERT2 mice (Figure 1C) after tamoxifen injection. The eIF3a mRNA and protein levels (Figures 1D, E) in tissues decreased significantly in eIF3a knockout mice. The expression of eIF3a protein in fat, lungs, skin, and spleen tissue was also detected by immunofluorescence staining (Supplementary Figure S2). The results of DNA, mRNA, and protein analyses showed that the eIF3a knockout mice model was efficaciously established.

### 3.2 Phenotypic characteristics of eIF3a knockout mice

**FIGURE 2:**
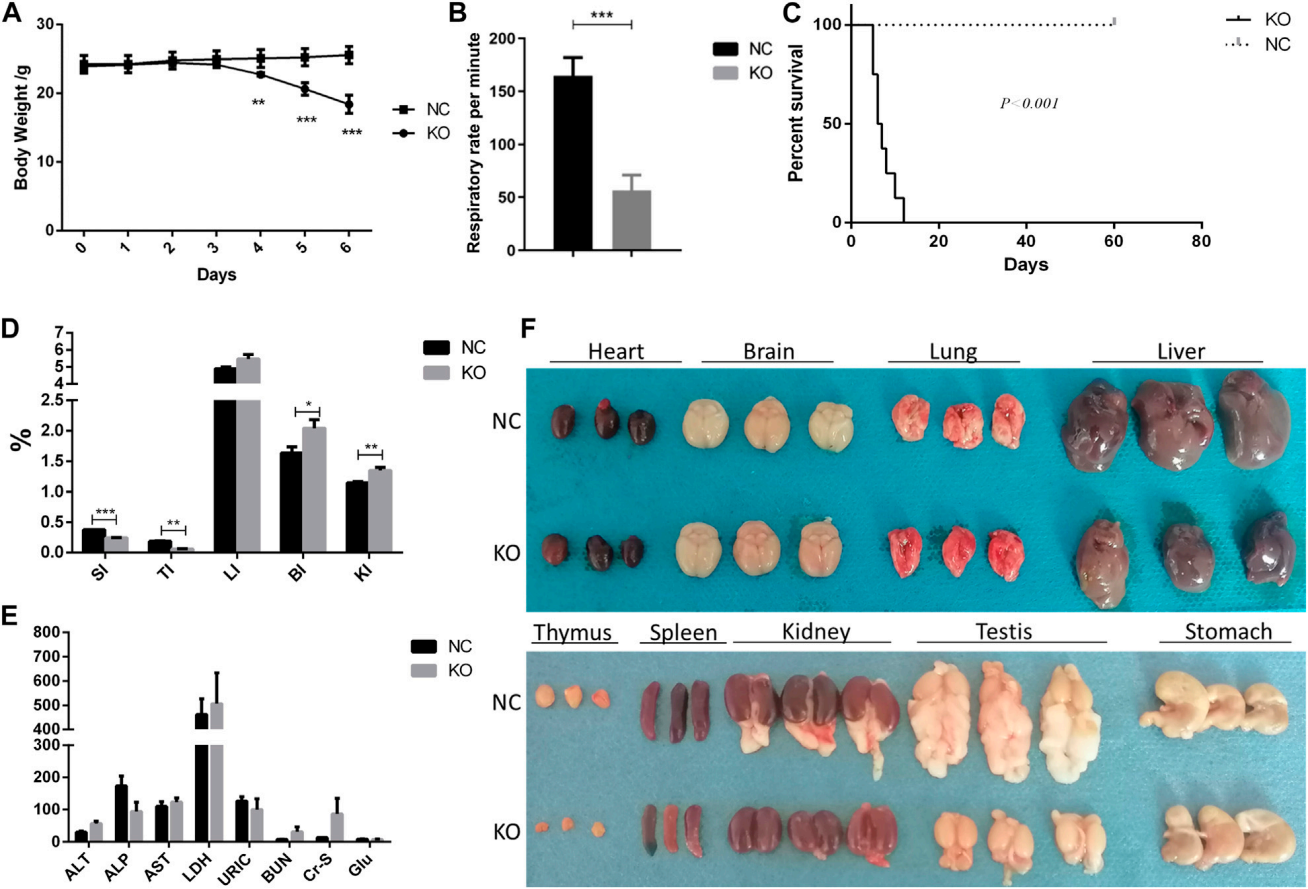
Phenotype of eIF3a conditional knockout mouse. **(A)** Body weight during the administration of tamoxifen (75 mg/kg) for 5 days. **p < 0.01; ***p < 0.001. **(B)** Respiratory rate in 24 h after the last dose of tamoxifen. ***p < 0.001; N = 6. **(C)** Survival curve of mice. Day 0 means 1 day before the first dose of tamoxifen. ***p < 0.001; N = 6. **(D)** Gut index (gut weight/body weight × 100%) of the mice. SI, spleen index; TI, thymus index; LI, liver index; BI, brain index. *p < 0.05; **p < 0.01; ***p < 0.001; N = 6. **(E)** Serum biochemical index of mice; ALT (U/L), alanine transaminase; ALP (U/L), Alkaline Phosphatase; AST (U/L), aspartate transaminase; LDH (mmol/L), lactate dehydrogenase; URIC (μmol/L), uric acid; BUN (mmol/L), blood urea nitrogen; Cr-s (μmol/L), serum creatinine; Glu (mmol/L), glucose. **(F)** Image of the gut of mice. Fat was attached to the testis and kidney.

The primary phenotypic characteristics of eIF3a knockout mice were studied. During a five-day induction by tamoxifen, the body weights of eIF3a knockout mice decreased significantly on the 4th day and continued to reduce in the next 2 days (Figure 2A). In addition, the respiratory rate decreased significantly in the eIF3a knockout group (Figure 2B). The median survival rate of eIF3a-deficient mice was 6.5 days; the survival rate of the control group was not reached (Figure 2C). No significant change was observed in H&E staining of the brain, heart, and liver, whereas the cell density was reduced in the thymus (Supplementary Figure S3). The size of the spleen, thymus, and genital and visceral fat was also lower in the knockout mice than in the control group (Figure 2F). Both cohorts of mice exhibited the presence of ingesta within their gastric compartments. The spleen (spleen weight/body weight) and thymus indexes were lower in the eIF3a knockout mice than in the control group. On the contrary, brain and kidney indexes were lower in the eIF3a knockout mice than those in the control, but the liver index was stable (Figure 2D). No significant difference was observed in the serum biochemical index (Figure 2E).

### 3.3 The differentially expressed proteins of fat tissue and bioinformatics analysis

**FIGURE 3:**
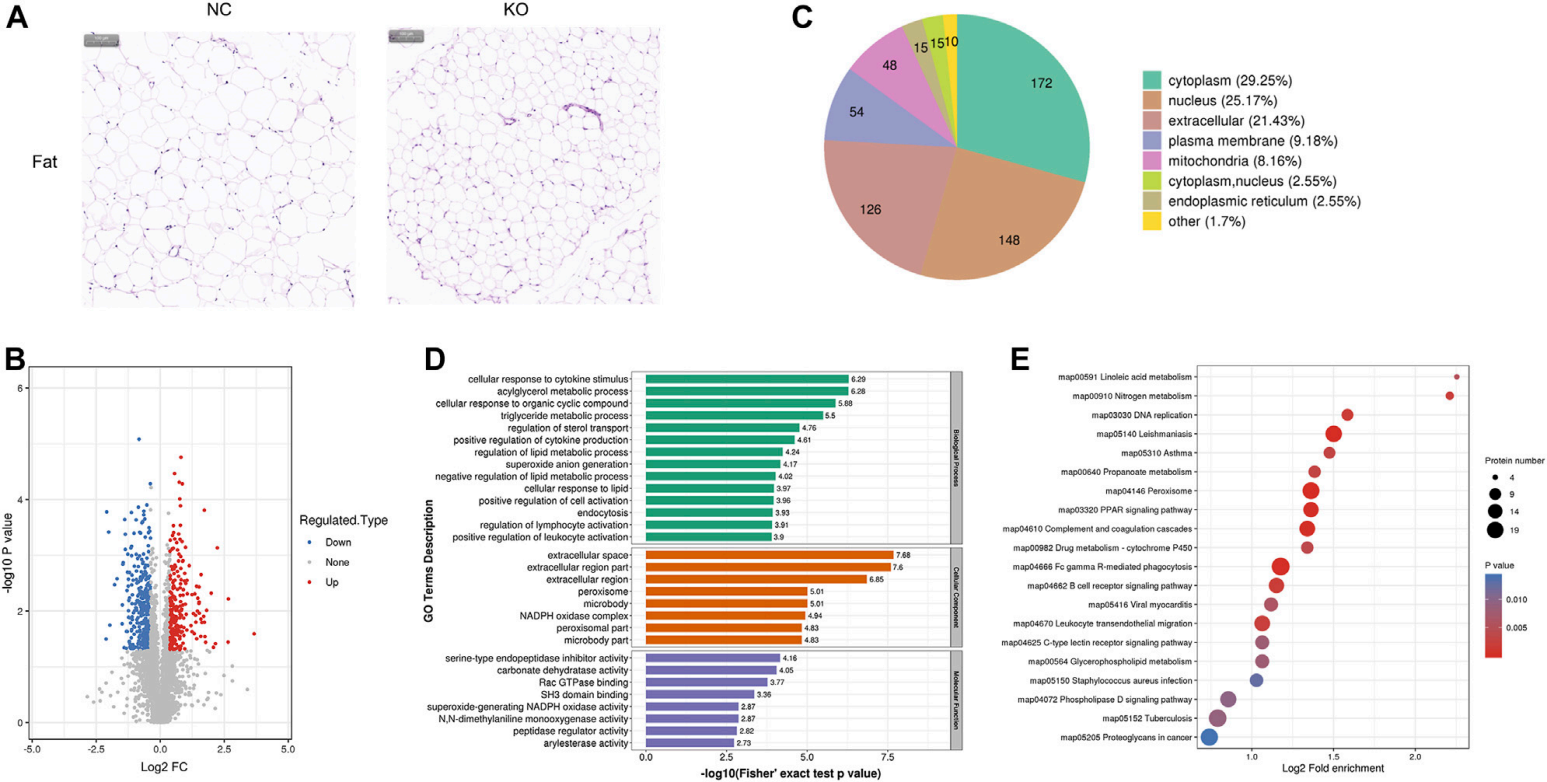
Histology and proteomics of fat in eIF3a knockout and control mice. **(A)** Hematoxylin and eosin of fat sections; the bar represents 100 μm. **(B)** Volcano plots show DEPs in eIF3a knockout mice compared to control; 346 proteins were downregulated and 242 proteins were upregulated. **(C)** Gene Ontology of DEPs in the category of cellular localization. **(D)** GO functional enrichment analysis of DEPs by Fisher’s exact test. The *x*-axis represented *p*-values. **(E)** KEGG enrichment of DEPs; circle size represents the number of DEPs in a pathway, and the color represents the *p*-value.

Significant phenotypic changes in fat, lungs, skin, and spleen tissues of eIF3a knockout mice were observed. Prior research on this subject matter has not been documented. Consequently, our study was initiated to investigate the downstream proteins of eIF3a and elucidate the molecular pathways that are correlated with novel functions in eIF3a-deficient mice. Histological staining showed a decrease in the size of adipocytes in eIF3a knockout mice (Figure 3A). A total of 588 DEPs were quantified by mass spectrometry, of which 242 were upregulated, while 346 were downregulated (Figure 3B). The subcell classification of DEPs (Figure 3C) was distributed as follows: cytoplasm (172), nucleus (148), extracellular (126), cell membrane (54), and mitochondria (48). GO enrichment analysis (Figure 3D) of DEPs showed that the cellular components were mainly enriched in extracellular-related regions, microbody, peroxisome, microbody part, and peroxisomal part. The top biological processes were enriched in the metabolism of lipid, acylglycerol, and triglyceride; cellular response to a cytokine stimulus, lipid, and organic cyclic compounds; regulation of sterol transport and lymphocyte activation; positive regulation of cytokine production, cell activation, and leukocyte activation; endocytosis; and superoxide anion generation. Molecular functions of DEPs were enriched in the activity of the serine-type endopeptidase inhibitor, peptidase regulator, and carbonate dehydratase and Rac GTPase and SH3 domain binding. The lipid metabolism function may have caused the phenotypic change in the fat of eIF3a knockout mice. KEGG pathway enrichment analysis (Figure 3E) presented a high level of enrichment in Fc gamma R-mediated phagocytosis, phagosome, proteoglycans in cancer, peroxisome, leishmaniasis, complement and coagulation cascades, leukocyte transendothelial migration, amebiasis, and signaling pathways, including PPAR, B-cell receptor, phospholipase D, and chemokine. According to the KEGG database, peroxisome, phospholipase D signaling pathway, B-cell receptor signaling pathway, and PPAR signaling pathway are oxidative stress-related pathways.

### 3.4 The differentially expressed proteins of lung tissue and bioinformatics analysis

**FIGURE 4:**
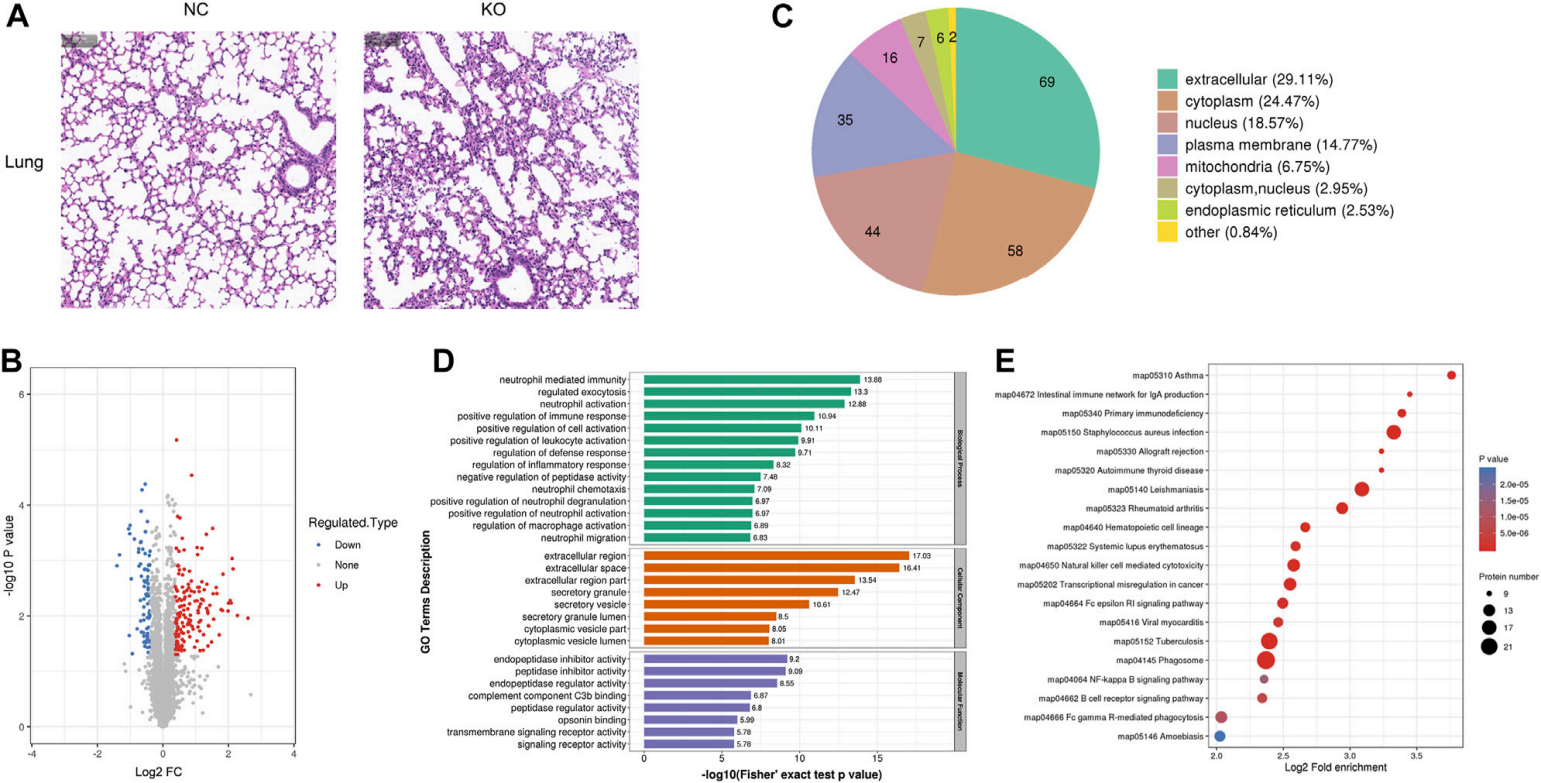
Histology and proteomics of the lungs in eIF3a knockout and control mice. **(A)** Hematoxylin and eosin of the lung sections; the bar represents 100 μm. **(B)** Volcano plots show DEPs in eIF3a knockout mice compared to control; 85 proteins were downregulated and 152 proteins were upregulated. **(C)** Gene Ontology of changed proteins in the category of cellular localization. **(D)** GO functional enrichment analysis of DEPs by Fisher’s exact test. The *x*-axis represents the *p*-values. **(E)** KEGG enrichment of DEPs; circle size represents the number of DEPs in a pathway, and the color represents the *p*-value.

The H&E staining of the lungs showed that the structure of the terminal bronchiole was clear and intact in both groups. However, the alveoli experienced shrinkage; the alveolar wall thickened with lymphocytic infiltration in eIF3a knockout mice (Figure 4A). Quantitative protein analysis revealed that there were 237 differentially expressed proteins, with 152 proteins exhibiting upregulation and 85 proteins exhibiting downregulation. (Figure 4B). Cell location and functional enrichment analyses were performed following the annotation of regulated proteins. Cell location analysis (Figure 4C) revealed that the DEPs were distributed in the extracellular (69), cytoplasm (58), nucleus (44), cell membrane (35), mitochondria (16), cytoplasm and nucleus (9), endoplasmic reticulum (6), and others (2). GO functional enrichment results were presented in biological process, cellular component, and molecular function (Figure 4D). The primary biological processes of DEPs were distributed in neutrophil-related functions, such as neutrophil-mediated immunity, neutrophil activation and degranulation, and neutrophil chemotaxis and migration. Furthermore, they were also involved in the regulation of immune response, defense response, leukocyte activation, inflammatory response, macrophage activation, and cell activation; regulated exocytosis; and the negative regulation of peptidase activity. The cellular component of DEPs was mainly enriched in the extracellular region, space, and region part; secretory granule, vesicle, and granule lumen; and cytoplasmic vesicle part and vesicle lumen. As for the molecular function of the DEPs, it was distributed in activity regulation, including endopeptidase inhibitor, peptidase inhibitor, endopeptidase regulator, peptidase regulator, transmembrane signaling receptor, and signaling receptor; complement component C3b; and opsonin binding. KEGG pathway (Figure 4E) enrichment analysis presented a high degree of enrichment in phagosomes, tuberculosis, *Staphylococcus aureus* infection, leishmaniasis, natural killer cell-mediated cytotoxicity, transcriptional misregulation in cancer, Fc gamma R-mediated phagocytosis, and rheumatoid arthritis. Two oxidative stress-related pathways, B-cell receptor and NF-kappa B signaling pathways, were also observed.

### 3.5 The differentially expressed proteins of skin tissue and bioinformatics analysis

**FIGURE 5:**
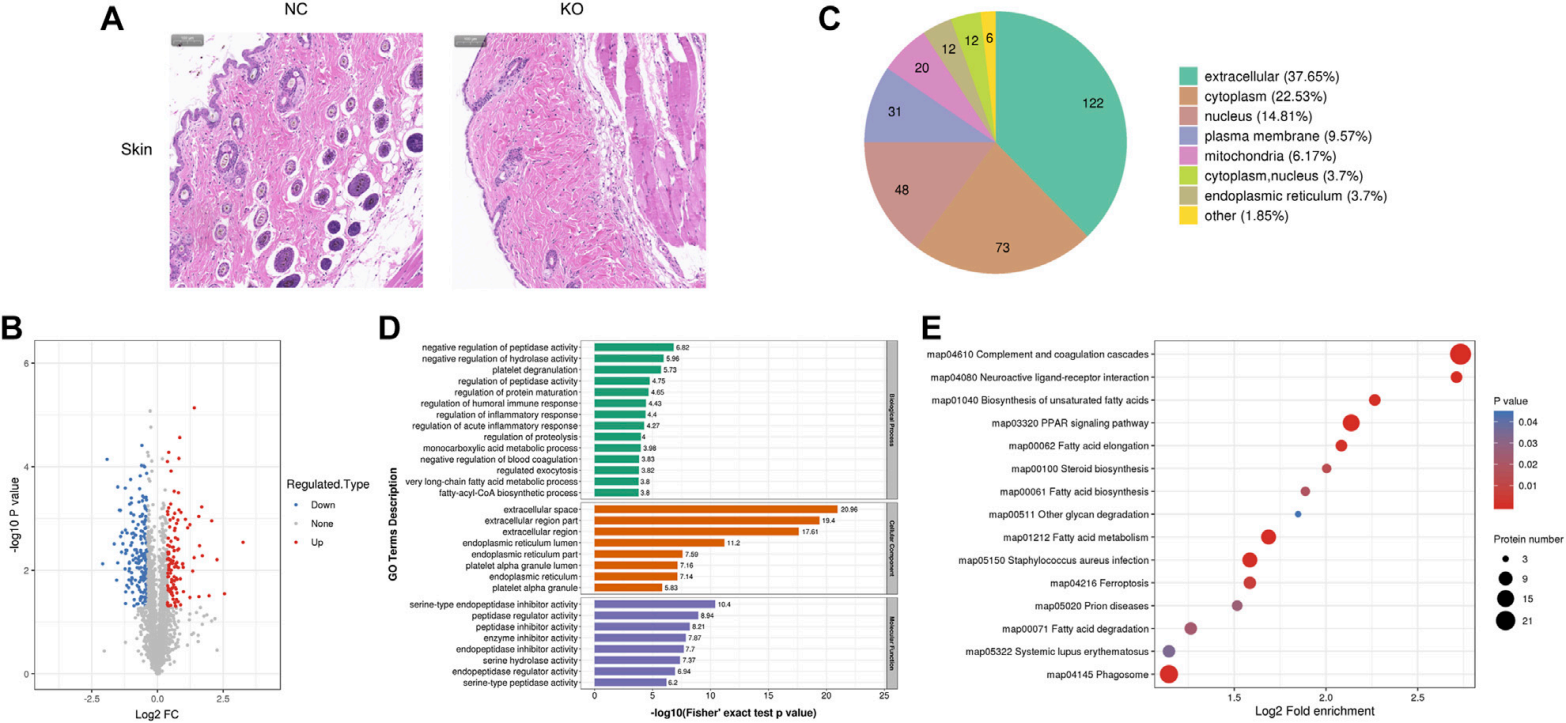
Histology and proteomics of the skin in eIF3a knockout and control mice. **(A)** Hematoxylin and eosin staining of the skin sections; the bar represents 100 μm. **(B)** Volcano plots show DEPs in eIF3a knockout mice compared to control; 204 proteins were downregulated, and 120 proteins were upregulated. **(C)** Gene Ontology of DEPs in the category of cellular localization. **(D)** GO functional enrichment analysis of DEPs by Fisher’s exact test. The *x*-axis represents the *p*-values. **(E)** KEGG enrichment of DEPs; circle size represents the number of DEPs in a pathway, and the color represents the *p*-value.

The histological result (Figure 5A) showed that the width of the epidermis, dermis, and subcutaneous fat was lower in eIF3a knockout mice than in the control. Hair loss and hair follicle cell reduction were also observed in eIF3a knockout mice. A total of 324 DEPs were observed, of which 120 were upregulated and 204 were downregulated (Figure 5B). Cell location analysis (Figure 5C) revealed that the downstream proteins of eIF3a in the skin were mainly located in extracellular (122), cytoplasm (73), nucleus (48), plasma membrane (31), and mitochondria (20). GO enrichment analysis found that the cellular component of DEPs was enriched in extracellular- and endoplasmic reticulum-related regions, platelet alpha granule lumen, and platelet alpha granule (Figure 5D). The biological process in GO analysis was mainly enriched in negative regulation of peptidase activity, hydrolase activity, and blood coagulation; regulation of peptidase activity, protein maturation, humoral immune response, inflammatory response, acute inflammatory response, exocytosis, and proteolysis; platelet degranulation; and monocarboxylic acid metabolic process. The molecular function of GO analysis was mainly enriched in mediating enzyme activity including serine-type endopeptidase, peptidase, endopeptidase, serine hydrolase, endopeptidase, and serine-type peptidase. The most enriched KEGG pathways (Figure 5E) were complement and coagulation cascade, PPAR signaling pathway, fatty acid metabolism, fat acid elongation, *Staphylococcus aureus* infection, phagosome, and ferroptosis. In these pathways, PPAR signaling pathway and ferroptosis are the two critical oxidative stress-related pathways.

### 3.6 The differentially expressed proteins of spleen tissue and bioinformatics analysis

**FIGURE 6:**
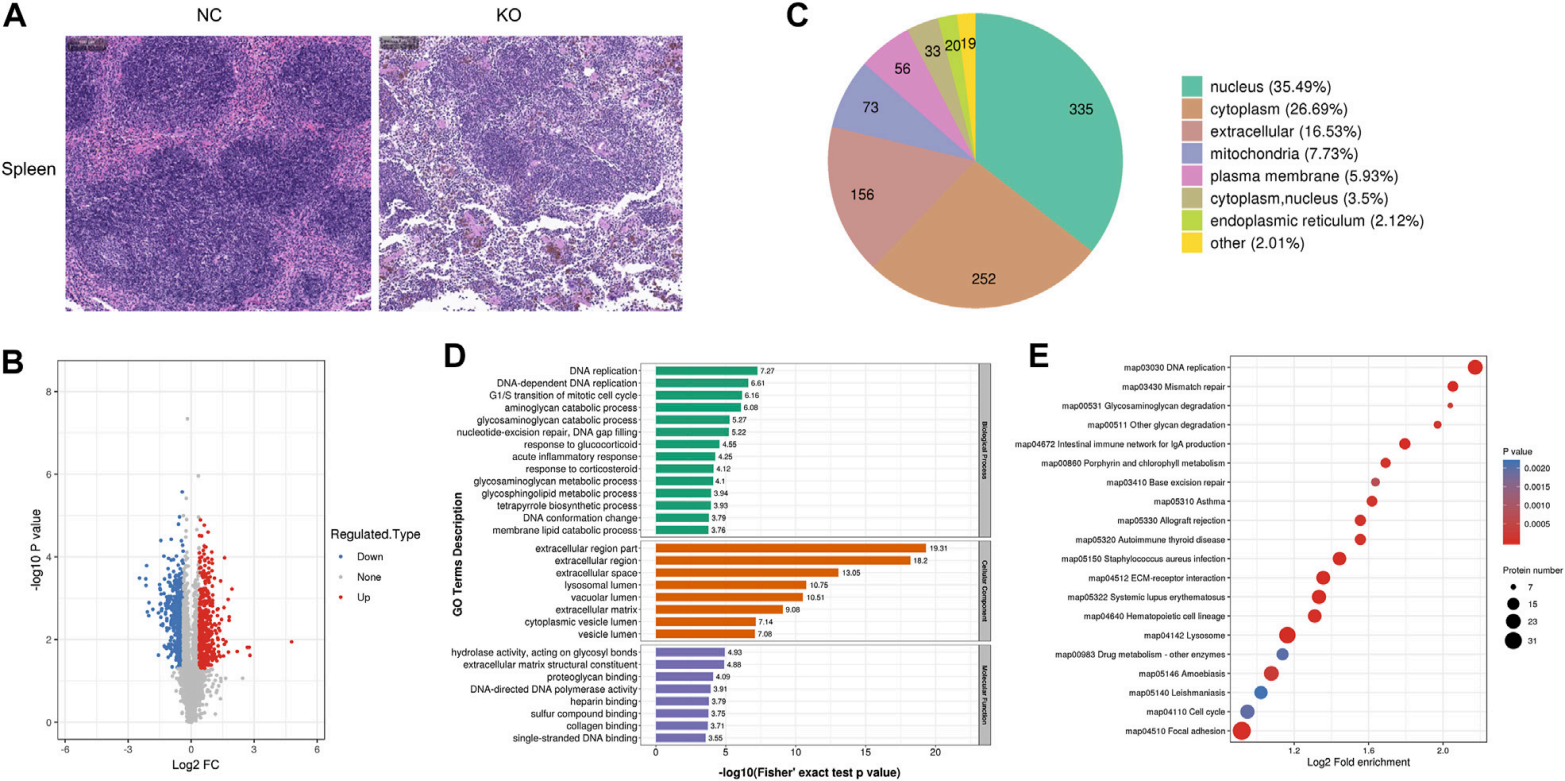
Histology and proteomics of the spleen in eIF3a knockout and control mice. **(A)** Hematoxylin and eosin staining of the spleen sections; the bar represents 100 μm. **(B)** Volcano plots show DEPs in eIF3a knockout mice compared to control; 526 proteins were downregulated, and 418 proteins were upregulated. **(C)** Gene Ontology of DEPs in the category of cellular localization. **(D)** GO functional enrichment analysis of DEPs by Fisher’s exact test. The *x*-axis represents the *p*-values. **(E)** KEGG enrichment of DEPs; circle size represents the number of DEPs in a pathway, and the color represents the *p*-value.

H&E staining of the spleen showed that the margins of the white pulp became blurred, and the density of cells in the white pulp decreased in the spleen of eIF3a knockout mice. Significant reductions of red blood cells in the spleen red pulp were also observed (Figure 6A). Compared with the control, 944 proteins were differentially expressed in spleens of eIF3a knockout mice, of which 418 were upregulated and 526 were downregulated (Figure 6B). Cell location analysis (Figure 6C) showed that downstream spleen proteins of eIF3a were distributed in nucleus (335), cytoplasm (252), extracellular (156), mitochondria (73), and plasma membrane (56). GO enrichment analysis indicated that the cellular component was enriched in extracellular-related regions, lysosomal lumen, vacuolar lumen, cytoplasmic vesicle lumen, and vesicle lumen (Figure 6D). Biological processes of GO enrichment analysis results showed that the DEPs were mainly enriched in DNA replication and repair; catabolic processes of aminoglycan, glycosaminoglycan, and membrane lipid; responses to glucocorticoid, corticosteroid, and acute inflammation; metabolic processes of glycosaminoglycan and glycosphingolipid; tetrapyrrole biosynthetic process; G1/S transition of the mitotic cell cycle; and DNA gap filling and conformation change. The molecular function of DEPs focused on hydrolase activity and acted on glycosyl bonds, extracellular matrix structural constituents, proteoglycan binding, DNA-directed DNA polymerase activity, and binding-related functions (heparin, sulfur compound, collagen, and single-stranded DNA). KEGG pathway enrichment analysis (Figure 6E) revealed that DEP pathways were focal adhesion, lysosome, amebiasis, ECM–receptor interaction, DNA replication, mismatch repair, *Staphylococcus aureus* infection, hematopoietic cell lineage, systemic lupus erythematosus, allograft rejection, and intestinal immune network for IgA production. The focal adhesion pathway was an oxidative stress-related pathway, of which 31 were upregulated and five were downregulated.

## 4 Discussion

This study reports the successful generation of eIF3a-floxed mice for the first time. The phenotype of these mice did not change compared to the wild-type mice. A recombinant PCR band was observed on eIF3a^flox/flox^, UBC-Cre-ERT2 mice after exposure to tamoxifen. We observed that eIF3a was knocked out in different tissues at different efficiencies. There were a series of phenotype changes in eIF3a knockout mice as compared to the control group. eIF3a was a lethal gene, even in fully developed individuals. Histological changes were observed in some tissues, especially in fat, lungs, skin, spleen, and thymus of eIF3a knockout mice. There were 588, 237, 324, and 944 differentially expressed proteins in fat, lungs, skin, and spleen, respectively. Bioinformatics analysis revealed new potential functionalities.

As the largest subunit of eIF3, eIF3a was reported to play a potential role in embryonic development (Liu et al., 2007). In our study, knockout eIF3a in 8-week-old mice led to death in a short time. This indicated that *eIF3a* was not only a development-related gene but also a crucial gene to sustain life. The respiratory rate decreased significantly in eIF3a knockout mice; the histological result also showed that the structures of the alveolus were destroyed. The study suggested that the death may have resulted from the destruction of lung function. The serum glucose level did not decrease, indicating that a lack of energy intake was ruled out as the cause of death. Stomach contents of knockout mice also supported this view. The histological results of fat and spleen indicated that eIF3a played a crucial role in lipid metabolism and immune response, which was identified in proteomics analysis.

eIF3a is involved in global mRNA translation, and it may regulate 15%–20% of the protein expression *in vitro* (Dong and Zhang, 2003). In this study, the number of differentially expressed proteins ranged from 237 to 944, of which only eight were shared by the four tissues (Supplementary Figure S4). In DEPs, the number of tissue-specific proteins was 369 in fat, 129 in the lungs, 177 in the skin, and 738 in the spleen. It suggested that the proteins regulated by eIF3a are spatio-dependent, and the role of eIF3a varies in different tissues.

As a translational initiation factor, eIF3a plays a crucial role in global RNA translation. GO enrichment results in this study also showed RNA-binding function. Meanwhile, eIF3a also regulated special mRNA translation involved in cancer incidence and development and affects the therapy of malignant tumors. However, the results were not consistent: patients with a higher expression of eIF3a have a better prognosis in lung cancer, esophageal cancer, ovarian cancer, cervical cancer, and bladder cancer, whereas opposite results were observed in patients with liver cancer and pancreatic cancer (Yin et al., 2018). Similar results were found in the Human Protein Atlas (http://www.proteinatlas.org/). These confusing results may be due to the proteins regulated by eIF3a being tissue-specific (Supplementary Figure S4). This study discovered new functions of eIF3a, including cytokine secretion, enzyme activity regulation, metabolic regulation, catabolic process, defense and inflammation response, and phagocytosis. It is promising for eIF3a to play roles in obesity, fatty liver, diabetes, infectious diseases, autoimmune diseases, tumor immunity, and inflammation. Further studies are needed to elucidate the detailed mechanism of these functions.

Cancer cells are typically exposed to hypoxic and malnourished conditions due to their high metabolic needs for proliferation (Ackerman and Simon, 2014). As an oncogene, eIF3a may play roles in stress. The expression of eIF3a varies with exposure to thermal stress, hypoxia stress, irradiation stress, nutrient starvation stress, and viral infections (Rodriguez Pulido et al., 2007; Dong et al., 2009; Lane et al., 2013; Trivigno et al., 2013; Ding et al., 2020; Malcova et al., 2021; Srivastava et al., 2021). However, its role in oxidative stress has been poorly studied. In cancer research, oxidative stress can result from external and internal factors, such as radiation and natural metabolic processes within the cancer cell, respectively. Oxidative stress can be used to activate certain oncogenes, which can further promote cancer growth (Hayes et al., 2020). Furthermore, other studies have shown that oxidative stress increases the risk of developing cancer and metastasis and resistance to treatment. In summary, oxidative stress plays a crucial role in cancer development by aiding cancer cells in their growth and spread and its absence potentially limiting cancer growth (Srivastava et al., 2021). In this study, the proteomics results indicate that eIF3a′s downstream proteins are enriched in oxidative stress-related pathways (KEGG database), such as nitrogen metabolism, peroxisome, drug metabolism cytochrome P450, pyruvate metabolism, PPAR signaling pathway, phospholipase D signaling pathway, B-cell receptor signaling pathway, ferroptosis, and focal adhesion. In addition, the DEPs in this study are involved in DNA replication and repair, inflammatory response, and phagocytosis, all of which are closely associated with oxidative stress (Babior, 2000; Reuter et al., 2010; Kryston et al., 2011). Stress granules (SGs) are dense aggregations in the cytosol composed of proteins and RNAs that appear when the cells are exposed to stress, and translation initiation is stalled. The proportion of DEPs in the cytoplasm was 29.25% in fat, 24.47% in the lungs, 22.53% in the skin, and 26.69% in the spleen, all of them taking an almost equal share. eIF3a was also widely expressed in the cytoplasm where the SGs were located. Numerous studies observed that eIF3a expressed in SGs was considered an SG-promoter protein (Kedersha et al., 2005; Van Treeck et al., 2018; Marmor-Kollet et al., 2020; Malcova et al., 2021). SGs increase in cancer and affect cancer development and therapy (Anderson et al., 2015). They together suggested that eIF3a may affect cancer phenotype and therapy by SGs. Our previous studies found that eIF3a increased the efficacy of cisplatin, anthracycline, and ionizing radiation, the well-known inducers of oxidative stress in cancers, by DNA damage repair (Yin et al., 2011b; Tumia et al., 2020; Chen J et al., 2021). Our work also found that it was involved in lipid peroxidation; the knockdown of eIF3a elevated the ROS level when cancer cells were exposed to cytotoxic antitumor drugs (unpublished). These studies indicate that eIF3a may be a bridge between oxidative stress and cancer, promoting elucidation of cancer development and therapy from the cellular process, metabolism, molecular signaling pathway, and immune response.

In conclusion, eIF3a conditional knockout mice were constructed for the first time, and eIF3a is still a lethal gene in adult mice. Phenotypic characteristics were found in eIF3a knockout mice. Tissue proteomics analysis was significantly different in tissues between eIF3a knockout mice and control mice, and the differentially expressed protein profiling was tissue-specific. A plethora of newly unveiled characteristics was identified from the proteomic results, especially oxidative stress-related function. Nevertheless, further studies are needed to unveil the detailed mechanism of regulation, such as polysome and ribosome sequencing in tissue-specific knockout mice at different ages. Also, new functions of eIF3a are needed to be explored further.

## Data Availability

Data is available *via* ProteomeXchange with identifier PXD041416.

## Supplementary material

Data Sheet 1 Supplementary Material relating to Figures and table.
