# Supplementary material for: Proteomic profiling of eIF3a conditional knockout mice
Source: Front Mol Biosci. 2023 Apr 19;10:1160063. doi: 10.3389/fmolb.2023.1160063 (PMC10154561; doi:10.3389/fmolb.2023.1160063)
Supplement: Supplementary file 1 [file DataSheet1.docx]

Supplementary Material

Proteomic profiling of eIF3a conditional knockout mice

Wei Zhuo^1,2^, Juan Chen^3^, Shilong Jiang^3^, Juyan Zheng^1,2^, Wei Li^1,2^, Zhibin Wang^1,2^, Hanxue Huang^1,2^, Mengrong Lei^1,2^, Jiye Yin^1,2^, Ying Gao^1,4*^, Zhaoqian Liu^1,2*^

*** Correspondence:** Zhao-Qian Liu, zqliu@csu.edu.cn; Ying Gao, Ying.gao@csu.edu.cn

# Supplementary Figures and Tables


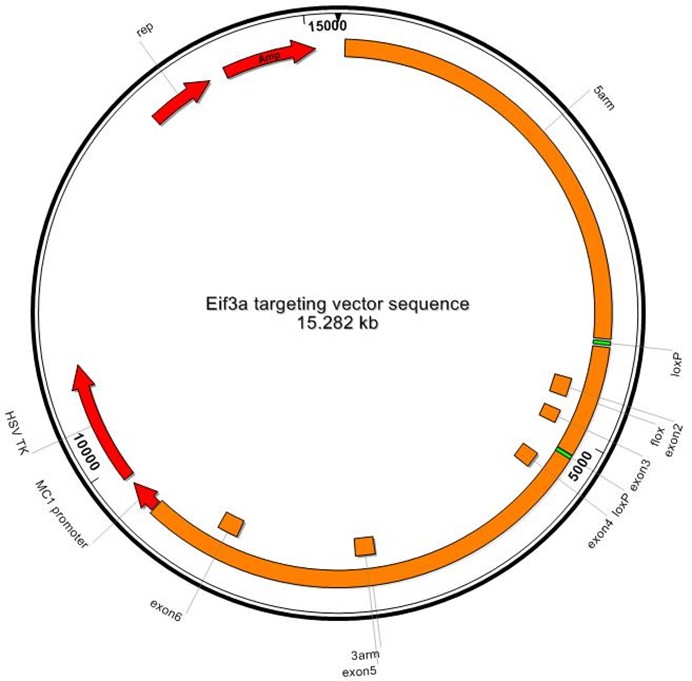


**Supplementary Figure 1.** Image of eIF3a targeting vector, green band indicate floxP site.


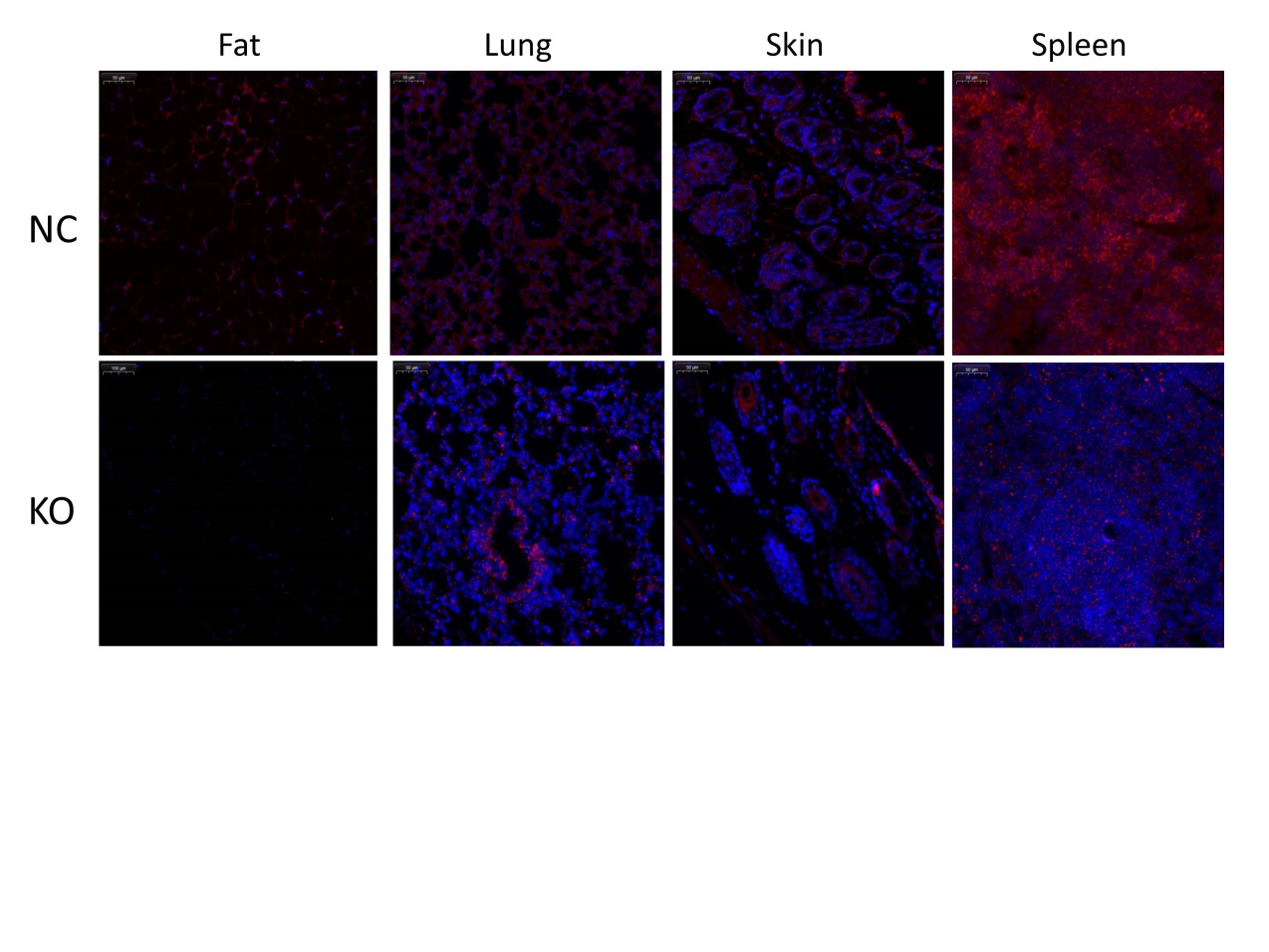


**Supplementary Figure 2.** Immunofluorescence of eIF3a in the fat, lung, skin, and spleen, the bar represents 50μm.


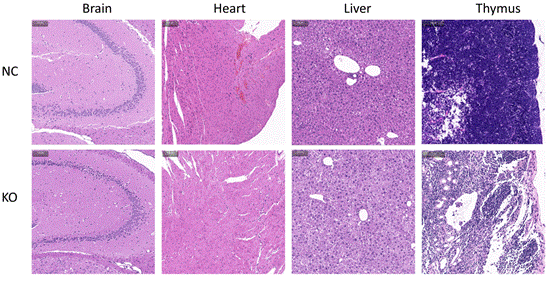


**Supplementary Figure 3.** Hematoxylin and eosin staining of brain, heart, liver, and thymus sections, the bar represents 100μm.


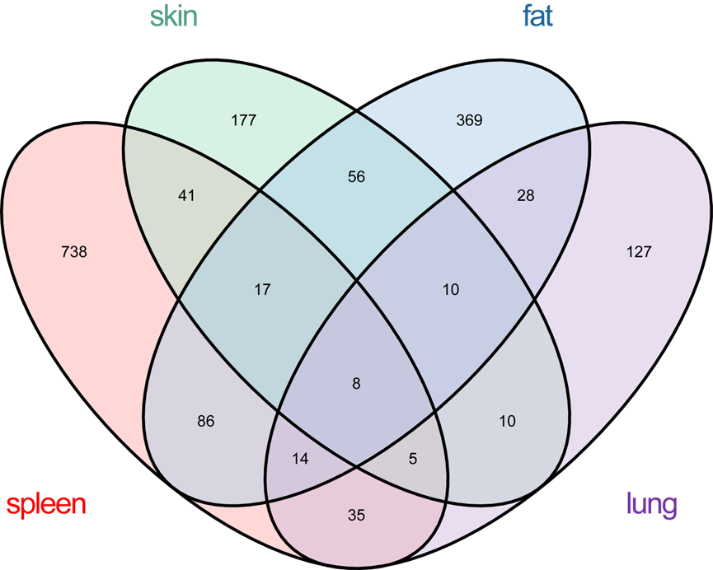


**Supplementary Figure 4.** Venn diagram of differently expressed proteins in fat, lung, skin, and spleen from eIF3a knockout mice comparing control mice.

Supplementary Table

**Table S1** Primer sequences for PCR and Real-Time qPCR

| Genes | | Primer Sequence |
| --- | --- | --- |
| eIF3a | Forward (flox) | CAGCTACAGAGTGATGCAGT |
|  | Reverse (flox) | TAGCAGGAGAACCCACTCTT |
| eIF3a | Forward (ko) | TTGATTGTGGGCGCTAGGAC |
|  | Reverse (ko) | ACACTGTCATTTTACCTTGTTGGGC |
| Ubc-Cre | Cre-Forward | ATTTGCCTGCATTACCGGTCG |
|  | Cre-Reverse | CAGCATTGCTGTCACTTGGTC |
|  | Positive Control-Forward | CAAATGTTGCTTGTCTGGTG |
|  | Positive Control-Reverse | GTCAGTCGAGTGCACAGTTT |
| eIF3a | Forward (for qPCR) | CCACTAGGGAGTTCGCTGAC |
|  | Reverse (for qPCR) | TCTGCCATGTTCTGTGCTTC |
| GAPDH | Forward (for qPCR) | TGGATTTGGACGCATTGGTC |
|  | Reverse (for qPCR) | TTTGCACTGGTACGTGTTGAT |
| β-actin | Forward (for qPCR) | GGCTGTATTCCCCTCCATCG |
|  | Reverse (for qPCR) | CCAGTTGGTAACAATGCCATGT |
